# Molecular Triggers of Yeast Pathogenicity in the Yeast–Host Interactions

**DOI:** 10.3390/cimb47120992

**Published:** 2025-11-27

**Authors:** Ortansa Csutak, Viorica Maria Corbu

**Affiliations:** 1 Department of Genetics, Faculty of Biology, University of Bucharest, Intrarea Portocalelor No. 1-3, 060101 Bucharest, Romania; ortansa.csutak@bio.unibuc.ro; 2 Research Institute of University of Bucharest (ICUB), Faculty of Biology, University of Bucharest, Hasdeu, No 7, 050568 Bucharest, Romania; 3 Research, Training and Consulting Center in Microbiology, Genetics and Biotechnology MICROGEN, University of Bucharest, Intrarea Portocalelor No. 1-3, 060101 Bucharest, Romania

**Keywords:** *Candida*, molecular pathogenicity, pH homeostasis, heat shock, hypoxia, hypercapnic state

## Abstract

*Candida* and other pathogenic yeast species, able to transition from non-invasive commensal organisms to invasive pathogens, are characterized by a high ability to adapt to stress conditions encountered in the human host, such as pH and temperature shifts, CO_2_ and oxygen level variations, and nutritional limitations. Although *Candida albicans* remains the main cause of *Candida*-related infections, non-*albicans Candida* (NAC) species, including *C. tropicalis*, *C. parapsilosis*, *C. krusei*, and non-*Candida* species such as *Yarrowia lipolytica*, *Candidozyma auris*, and *Nakaseomyces glabratus*, are gaining clinical importance. These species exhibit diverse mechanisms of pathogenicity, including morphological transition, modulation of gene expression pathways (cAMP-PKA/MAPK, Hsp, calcineurin, GlcNAc-mediated signaling), cell wall remodeling, post-translational reprogramming, biofilm formation, antifungal resistance, and enzyme secretion. *C. albicans* exhibits high morphological and metabolic plasticity for survival across body niches. *N. glabratus* and *C. tropicalis* show strong azole resistance and biofilm formation, while *C. parapsilosis* and *C. krusei* pose risks through surface adhesion and treatment resistance. *C. auris* stands out for heat tolerance, multidrug resistance, and outbreak potential. *Y. lipolytica*, though rare, forms persistent filamentous biofilms in critical care settings. *Cryptococcus neoformans* remains a life-threatening pathogen capable of immune evasion and crossing the blood–brain barrier. This review compares molecular mechanisms of pathogenicity across these fungi, emphasizing environmental adaptation, conserved and species-specific responses, and potentially highlighting targets for therapeutic management.

## 1. Introduction

The genus *Candida* comprises a diverse group of opportunistic fungal pathogens that have evolved elaborate mechanisms to adapt to and exploit the diverse and dynamic environmental niches within the human host, thus facilitating the transition from commensal microorganisms to invasive pathogens. While *Candida albicans* remains the most prevalent species, several non-*albicans Candida* (NAC) species, such as *C. tropicalis*, *C. parapsilosis*, *C. krusei*, and non-*Candida* yeast species, namely *Nakaseomyces glabratus* (formerly Candida glabrata), *Yarrowia lipolytica* (formerly *Candida lipolytica*), or *Candidozyma auris* (formerly *Candida auris*), have become increasingly recognized and studied for their clinical relevance, particularly in immunocompromised hosts, as well as for their ability to spread rapidly and cause outbreaks. *C. abicans* is known to colonize the gastrointestinal tract and the vaginal and oral mucosae as a commensal organism, while causing candidiasis under changes in environmental factors and microbiota composition or in immunocompromised patients [1,2]. Impaired immunity, as well as prolonged stays in hospital or intensive care units, represent the main factors for invasive yeast infections, most of them comprising *Candida* species (*C. albicans* > *N. glabratus > C. tropicalis > C. parapsilosis > C. krusei*), with *C. albicans* encountering more than 50% in some cases [3]. The ability to adapt its morphology, metabolism, and stress signaling pathways to environmental stimuli associated with various human body niches makes *C. albicans* one of the most versatile fungal pathogens.

*N. glabratus* is commensal on human epithelia (skin, gastrointestinal tract, genital area) and causes infections when spreading to the bloodstream, urinary tract, or oral cavity. Epidemiological studies—still largely reported under its former name, *C. glabrata*—identify this species as the second most common cause of invasive candidiasis in the United States and Northwestern Europe, with high morbidity and mortality (up to 60%) [4]. Unlike *C. albicans*, which exhibits polymorphism and can transition between yeast, pseudohyphal, and hyphal forms, *N. glabratus* is a strictly unicellular yeast that does not form true hyphae. However, its resistance to azole treatment and high capacity to form biofilms and adhere to biotic and abiotic surfaces represent a serious threat [5].

*C. tropicalis* seems to be prevalent in tropical and sub-tropical regions and presents wide genetic variability among strains, with gene transfer between populations from different regions [6]. *C. tropicalis* is described as having similar behavior as *N. glabratus* in terms of adherence (to epithelial and endothelial cells), biofilm production, and antifungal resistance to azole derivatives and, additionally, to amphotericin B and echinocandins [7]. Moreover, its pathogenicity shows resemblance to *C. albicans*, associated with filamentous growth, adaptation to environmental variations, and synthesis of enzymes such as aspartyl proteinases associated with virulence [5,8].

The ability of *C. parapsilosis* to colonize the skin, nails, gastrointestinal tract, and reproductive tract without symptoms, as well as its presence in household or healthcare wastewater, facilitates its spread and the emergence of opportunistic infections [9]. *C. parapsilosis* is one of the main species affecting immunocompromised patients and presents a high ability to survive stress conditions related to human hosts (temperature, pH, nutritional shifts) and to form biofilms on the surface of medical devices as a mixture of yeast and elongated, hyphal-like cells [10].

Although *C. krusei* (teleomorph *Pichia kudriavzevii*) is less virulent than *C. albicans*, causing superficial oral or vulvovaginal infections, it represents the main pathogen encountered in candidemia, endocarditis, and arthritis associated with high mortality rates (up to 58%). *C. krusei* is resistant to antifungal agents (fluconazole), has a high capacity to adhere to abiotic surfaces (polyethylene, polyvinylchloride, or glass), and secretes enzymes involved in virulence (aspartyl proteinases, hemolysins, proteases, etc.) [11], representing one of the main species in nosocomial infections.

Lastly, although rather recently reported in 2009, *C. auris* is one of the most threatening pathogens causing outbreaks worldwide, with mortality rates as high as 60% [12]. Besides its multidrug resistance, it has a remarkable ability to adapt to prolonged and increased stress conditions (temperature, reactive oxygen species, dryness, salt, nutritional restraints) due to elaborated transcriptional mechanisms and to survive in human niches not ordinarily colonized by *Candida* spp., such as blood or hair follicles [13].

Formerly known as *C. lipolytica*, *Y. lipolytica* is mainly studied as a non-conventional yeast species harboring strains with high metabolic versatility and numerous industrial and biotechnological applications [14]. *Y. lipolytica* is a rare opportunistic pathogen, especially in the case of immunocompromised and critical patients from intensive care units. The main causes of *Y. lipolytica* pathogenicity are related to its ability to adhere long time on the catheter surface (about 168 h), forming biofilms with filamentous growth, and to produce hydrolytic enzymes that attack host cells [15,16].

Other life-threatening pathogenic fungi include species of the genus *Cryptococcus*, notably *Cryptococcus neoformans* (serotype A and D) and *Cryptococcus gattii* (serotype B and C). Infections usually occur through the inhalation of basidiospores or desiccated yeast cells from environmental sources (trees, birds), which cause pulmonary disease that can disseminate to the central nervous system, causing cryptococcal meningitis. Although human-to-human transmission is not demonstrated, *Cryptococcus* spp. is a high-ranked fungal pathogen due to its capacity to escape the host immune system, to survive within macrophages, to survive temperature variations, and to return to the environment in a non-pathogenic state, thus restarting the cycle [17,18].

The present review aims to offer a comparative perspective upon some of the most important molecular mechanisms related to pathogenicity in *C. albicans*, NAC species, and non-*Candida* species (*C. auris*, *N. glabratus*, *Y. lipolytica*, and *C. neoformans*), that are triggered in response to changes in environmental factors specific to human hosts (pH, temperature, CO_2_, oxygen limitation, nutrient shifting), highlighting both conserved and species-specific adaptive strategies, regulatory pathways, and their implications for fungal pathogenicity and host interaction.

## 2. Adaptation to pH Shift

### 2.1. Alkaline pH

*C. albicans* can adapt to pH variations characteristic of host organisms, in alkaline (tissues, blood) or acidic (gastrointestinal, urinary tract) environments. In *C. albicans*, cell response to alkaline pH is mediated by two pathways: Rim101, which is preponderant, and cAMP-MAPK kinase.

**Figure 1:**
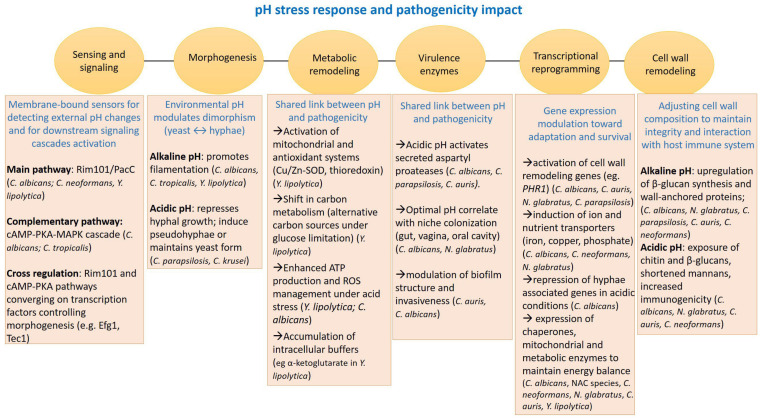
Integrated pH stress response pathways linking environmental pH sensing to pathogenic yeast morphogenesis and metabolism.

The Rim101 pathway debuts with the hyperphosphorylation of the arrestin-like protein Rim8, followed by the endocytosis of the plasma membrane complex (proteins Dfg16, Rim9, and Rim21) [19] and the recruitment of the Rim13 and Rim20 proteases, which activate Rim101 by cleavage of its repressor C-terminal domain [1,20,21]. This triggers the transcriptional activation of numerous genes involved in morphological and metabolic processes as follows. (1) The over-expression of genes encoding cell-wall-associated proteins: *PHR1* (glycosylphosphatidylinositol-anchored β(1,3)-glucanosyltransferase) with a role in cell wall remodeling is expressed 60-fold more in pH 8.0 compared to pH 4.0, *PRA1* (fibrinogen-binding protein), which are expressed 278-fold more, while *ECE1*, *HWP1*, *HYR1*, *IHD1*, *RBT1*, *SAP4*, and *SAP6* encoding cell-wall-associated proteins with role in filamentation are expressed 31-fold in pH 8.0 compared to pH 4.0. (2) The up-regulation of genes responsible for iron transport: *FTR1/2* and *FTH1* (high-affinity iron permeases), *FET34* (multicopper oxidase), *CFL1*, *FRE2*, *FRE7*, *FRE9*, and *FRP2* (ferric reductases), *SMF3* (vacuolar iron transporter), and *ARN1/SIT1* (iron-siderophore binding protein), respectively, involved in membrane transport of other microelements: phosphate (*PHO84* and *PHO89*), copper (*CTR1*), manganese (*SMF1*), sodium (*ENA2*), and zinc (*ZRT1*). (3) Transcription of 29 ORFs for ribosomal biosynthesis, 3 ORFs required for protein glycosylation, and 10 ORFs important for protein trafficking [22,23] (Figure 1).

The cAMP-dependent MAPK kinase pathway is also involved in the response of *C. albicans* cells to pH modifications. At neutral pH, the Ras1 GTPase activates Cyr1, enabling protein kinase A (PKA) to activate the transcriptional factor Efg1. Subsequently, Efg1 induces the expression of genes involved in hyphae formation (*HWP1*, *ECE1*, *SAP*s) as well as in the expression of other transcriptional regulators (*EED1*, *TEC1*, and *CRZ1*) with a role in filamentation and hyphal development [24,25,26].

However, other proteins were identified as being involved in alkaline pH response in *C. albicans*. Thus, the mass spectrometry study of the cell wall revealed the presence of Eno1 enolase with transglutaminase activity and a major role in yeast–hyphae transition, which also represents the main antigen found in patients with candidiasis [27].

In contrast with *C. albicans*, *N. glabratus*, and *C. guilliermondii*, *C. auris* showed the highest survival rate at extreme pH (13.0) and under combined stress conditions comprising 47 °C temperature [28].

Biofilm formation plays a key role in *C. albicans* and *C. tropicalis* virulence and pathogenicity. Barbosa et al. [29] demonstrated that pH ranging between 5.9 and 7.0 does not seem to have an important impact on biofilm formation and planktonic growth in vitro, in experiments with artificial saliva and artificial urine, respectively. On the contrary, the number of cultivable *C. tropicalis* cells from biofilm and the biofilm mass registered a maximum at alkaline pH (7.0, 8.0) [30]. However, *C. tropicalis* formed biofilms even under acidic pH (3.0, 4.0), thus enhancing its ability to adapt to various niches with different pH values. The pH also seems to affect the structure of the biofilm that was present as a monolayer at pH 4.0 and 7.0, and as a compact multilayer at pH 8.0. Moreover, the invasion capacity was maximal at alkaline pH (8.0), being correlated with predominant filamentous growth [30] (Figure 1).

*C. neoformans’* adaptation to alkaline pH is correlated with niche colonization. Thus, in mammalian hosts, the pH response requires the expression of the genes *CNA1* and *ENA1* encoding calcineurin, respectively, a fungus-specific sodium or potassium P-type ATPase. Clacineurin is a serine/threonine Ca^2+^-calmodulin-dependent protein phosphatase highly conserved in eukaryotes. In *C. neoformans*, calcineurin is essential for calcium signaling, survival under alkaline pH and high temperature conditions, sexual development, antifungal resistance, and virulence [31]. Interestingly, the *ENA1* gene appears to be upregulated only in *C. neoformans* cells from infections affecting the human cerebrospinal fluid (CSF), along with *RIM101* and other seven genes with role in cell wall structure and function (*AGS1*, *EBG1*, *CDA1*, *FKS1*), host immune response (*CFO1*) and iron uptake (*CIG1*, *SIT1*) [32] (Figure 1).

As described for *C. albicans*, in the case of *Y. lipolytica*, the cellular response to alkaline pH is primarily regulated by the Rim101/PacC signaling pathway, but the entire mechanism remains insufficiently understood. Sekova et al. [33] proved that under alkaline stress, *Y. lipolytica* adapts primarily by remodeling its cell wall proteome, by increasing chaperone levels and mitochondrial proteins, including voltage-dependent anion channel (VDAC) or malate dehydrogenase, and also by shifting carbohydrate metabolism towards an energy-maintaining strategy. Moreover, the proteomic response is significantly different when cells are exposed to alkaline stress or alkaline stress associated with high temperature (pH 9.0 and 38 °C), assuring a cross-adaptation mechanism, which includes protection against lipid peroxidation and protein destruction [33,34] (Figure 1). *Y. lipolytica* cell morphology varies from oval to pseudohyphae or hyphae form as a direct response to environmental conditions, and it is presumed to be a coping mechanism for reaching nutrients [35]. The study by Shu et al. [36] proved that there is a strong correlation between *Y. lipolytica* cellular response to alkaline pH and the filamentation process, which is mediated by the Rim101/PacC signaling pathway in association with Msn2/Msn4-*like* transcription factor, YlMhy1. YlMhy1 and YlMsn4 are the only Msn2/Msn4 proteins in *Y. lipolytica*. According to Wu et al. [6], each of them has distinct roles in cellular regulation in response to different stress conditions: YlMhy1 is a key positive regulator for filamentation and invasive growth, while YlMsn4 acts in acidic conditions, and it might be a species-specific adaptation in *Y. lipolytica*. Between YlRim101 and YlMhy1, there is a direct connection; YlRim101 (activated in alkaline conditions and undergoing an autoregulation mechanism) partially regulates the expression of Mhy1. Shu et al. [36] indicate that, since the expression of *MHY1* is not completely inhibited in Yl*RIM101*-deficient strains, its regulation might be mediated through YlRim101-dependent and independent mechanisms.

According to their study, this signaling pathway intervenes in the regulation of specific genes. YlRim101 alone upregulates Yl*PHR1* and Yl*CRH12*, homologues to Ca*PHR1* and Ca*PHR2*, but has no influence on Yl*KRE6*, which is involved in cell wall biosynthesis in alkaline conditions as described for *C. albicans* [36,37]. However, in association with YlMhy1, YlRim101 intervenes in the regulation of five adhesin-like encoding genes, which are drastically downregulated in *mhy*-deficient strains. Up to this point, no gene associated with dimorphism has been found to be regulated solely by YlMhy1. However, YlMhy1 directly interferes with their expression due to its binding motif WNAGGGG [6] placed in the upstream intergenic region of eight cell wall protein genes.

### 2.2. Acidic pH

According to Ikeda et al. [38], the acidic pH has a strong impact on the survival of *Candida* yeast or hyphal cells. Under laboratory conditions, at pH 5.0, *C. albicans* and *C. tropicalis* cells were able to form hyphae, while *C. parapsilosis* formed pseudohyphae. Moreover, filamentous cells from these species, transferred into a highly acidic medium (pH 1.0), survived only for 4 h. *N. glabratus* was able to survive in acidic pH in yeast form, but at low rates (4.8%). Under the same conditions, the survival rates in yeast form compared to hyphae were slightly higher for *C. albicans*, *C. tropicalis*, and *C. parapsilosis*.

The inhibition of hyphal growth in *C. albicans* exposed to an acidic environment has several proposed explanations. First, the Rim101 pathway is inactivated due to the existence of the Rim101 protein in a non-cleaved, non-activated form. On the other hand, Rai et al. [39] demonstrated that, in the presence of gut conditions, the transcription of genes *EFG1*, *UME6*, *ROB1*, *TEC1*, and *FLO8* encoding regulators of hyphal growth, filamentation, biofilm formation, and virulence is repressed. Also, it seems that the gene *NRG1* is expressed 3-fold at pH 4.0 compared to pH 8.0, repressing the expression of *ECE1* responsible for the synthesis of candidalysin involved in adhesion, biofilm formation, and filamentation [22]. In addition, under acidic conditions, the transcription factor Rfg1 regulates Brc1 and suppresses the expression of genes related to hyphae development, *PHR1* and *ENA2* (sodium transporter) (Figure 1) [40].

In vitro experiments showed that at pH 4.0, *C. albicans* transcription factors *Stp2* (involved in regulation of expression of amino acid permease genes) and *Dal81* (a positive regulator of expression of genes for nitrogen-degradation from amino acids) are involved in medium alkalinization, amino acid uptake and promotion of hyphae formation which enables the escape of *Candida* cells from macrophage-mediated killing [41].

pH values of 2.0 and 4.0 determine cell wall remodeling in *C. albicans*, respectively, chitin and β-glucan exposure, which finally induces a higher proinflammatory cytokine response in the colonized tissue [42]. Also, the outer layer of the cell wall presents reduced mannan fibril length. Strong acidic conditions (pH 2.0) determine the de novo synthesis of chitin, reduced expression of the gene *CHT2*, followed by chitin exposure on the cell surface. This remodeling of the cell wall is responsible for β-glucan exposure. The process of unmasking of β-glucan is also found for *C. tropicalis*, *C. dubliniesis* [42], and *C. krusei* [1]. In the case of *C. krusei*, it was observed that when lactate is used as a carbon source in culture medium with the overall pH value of 4, *C. krusei* exhibits an enhanced growth rate compared to when glucose is used [43]. These results suggest that *C. krusei* can metabolize lactate efficiently under acidic conditions, which might represent a survival advantage in lactic acid-rich environments such as the vaginal niche. However, when exposed to higher concentrations of lactic acid, *C. krusei* growth is inhibited, which might indicate some differences regarding the intensity of cellular response to dual stressors such as pH and the presence of an antimicrobial compound such as lactic acid. Moreover, the study performed by Fakhruddin et al. [44] showed that *C. krusei* pH response influences its colonization and virulence in the oral cavity. In severe early childhood caries lesions, *C. krusei* emerged as the predominant non-*albicans* species, particularly within acidic niches from occlusal dentine samples (pH < 7). Multispecies *Candida* communities containing *C. krusei*, *C. tropicalis*, and *C. albicans* were detected exclusively in acidic lesions, whereas no yeast cohabitation occurred in neutral or alkaline environments. The persistence of *C. krusei* in such low-pH conditions indicates a similar adaptive metabolism to that described for other *Candida* species. Most probably, its persistence is associated with biofilm formation in the deep carious dentine, where the microenvironment is characterized by low salivary flow and high carbohydrate availability [43,44].

According to Dev [45], the gene *HSP30/31* encoding the small weight heat shock protein Hsp30/31, with the main role in protein homeostasis in *Candida*, is upregulated under acidic pH as well as under iron deprivation.

The synthesis of some virulence factors in acidic pH media is also a key factor in *Candida* pathogenicity. In this respect, the secreted aspartyl proteinases (Saps), key enzymes involved in virulence and pathogenesis of *Candida* cells, have an optimal proteolytic activity at acidic to neutral pH values [46]. Thus, a pH ranging from 3.0 to 5.0 correlates with high activity of Saps 1, 2, and 3 from *C. albicans* and Saps 1, 2, and 3 from *C. tropicalis*. Nevertheless, *C. albicans* also synthesizes Saps 4, 5, and 6 under slightly acidic to neutral conditions (pH 5.0 to 7.0), promoting the colonization of a wide variety of tissues [47]. High Saps activities were reported for *C. parapsilosis* at pH 3.0–4.0, respectively, for *C. auris* and *C. duobushaemulonii* at pH 4.0–5.0. In the case of *C. haemulonii* clade species, particularly for *C. auris* and *C. duobushaemulonii*, the Sap activity seems to be also correlated with a temperature of 37 °C [48].

While alkaline stress response in *Y. lipolytica* involved mainly transcriptional reprogramming coupled with cell wall remodeling and genetic dimorphism, in acidic conditions, the response relies more on organelle function and membrane-associated chaperones (Hsp12) [34]. Although it exhibits high tolerance to alkaline environments and high salt concentrations, *Y. lipolytica* is considered to prefer a low pH [49]. Under acidic stress, the proteome of *Y. lipolytica* is significantly remodeled by upregulation of proteins that maintain intracellular pH. Cytoplasmic acidification triggers membrane hyperpolarization, enhances ATP synthesis, and stimulates ROS production. To counteract these problems, the main response is an increase in the levels of antioxidant proteins such as Cu/Zn-SOD or thioredoxin and chaperons. Moreover, the VDAC levels also rise in order to support metabolite exchange between the cytosol and mitochondria [33,34]. Similar to *C. albicans*, in the case of *Y. lipolytica*, the acidic pH is not correlated with cellular dimorphism as described for other dimorphic fungi such as *Ustilago maydis* or *Trichosporon cutaneum* [50,51,52]. As mentioned previously, *Y. lipolytica’s* tolerance to an acidic environment might be mediated by a species-specific transcriptional factor YlMsn4, which is upregulated at low pH values [6]. However, its downstream gene target and its role in genetic dimorphism remain to be determined.

### 2.3. pH Homeostasis

*C. albicans* has a high ability to adapt to host-related stress conditions, the acidic pH alkalinization representing an important mechanism for survival, assimilation of micronutrients, and correct protein and cell functions. The plasma membrane H^+^-ATPase Pma1of *C. albicans* plays a significant role in pH homeostasis. Pma1 is regulated by glucose and acts in the alkalinization of the external environment by extruding protons from the cell. The C-terminal domain is mainly responsible for filamentation and maintenance of vacuolar morphology, its massive truncation (with 38 amino acids) leading to reduction in Pma1 levels (up to 90%), ATPase activity (75%), diminished growth rates, and vacuolar fragmentation [53].

*N. glabratus* does not produce SAPs, but instead it produces yapsin, a member of a family composed of 11 cell surface GPI-linked aspartyl proteases (CgYps1-11), highly similar to *S. cerevisiae* yapsins. From these, the synthesis of Yps1, Yps4-5, and Yps8-11 is upregulated upon *N. glabratus* internalization by macrophages and is essential for cell survival, while Yps1 and Yps7 are involved in cell wall remodeling [54]. Additionally, Yps1 is required for assuring pH homeostasis in the presence of acidic pH by regulating the proteolytic processing of CgPma1 (the ortholog of Pma1 from *C. albicans*) [55].

The putative acetate/ammonia transporters Ato1 and Ato5, respectively, the acetyl-coenzyme A (CoA) hydrolase Ach1 involved in acetyl-CoA metabolism for fatty acids and cholesterol synthesis, are also essential for medium alkalinization in *C. albicans*, in correlation with glucose-deprivation and usage of alternative carbon sources [56]. Moreover, in vitro experiments revealed that mutations affecting the *ATO1* and *ATO5* genes reduced ammonium release from the cells and impaired hyphae formation [57].

Regarding the pH homeostasis in *C. krusei*, comparative genomic and functional analyses performed by Akita et al. [58] *C. krusei* NBRC1664 and NBRC1395 showed that the first one exhibited superior adaptability to acidic conditions. This can be explained by an intact and efficient High Osmolarity Glycerol (HOG) MAPK pathway. More precisely, *C. krusei* NBRC1664 has the essential upstream components of the pathway (Opy2, Sln1, and Cdc24), which are absent in the *C. krusei* NBRC1395 strain [58,59]. Opy2 and Sln1 are transmembrane sensors that initiate MAPK signaling in case of osmotic or ionic stress, while Cdc24 is an activating factor for Cdc42 involved in cell division and polarity [60]. Another hypothesis is that the alcohol dehydrogenase-like family of proteins (ADHF1 and ADHF2) is also involved in pH stress adaptation through detoxification and redox balance. The same *C. krusei* NBRC1664 strain presented higher levels of ADHF1 and ADHF2 proteins involved in the cellular protection against toxic compounds such as furfural and hydroxymethylfurfural [58].

Another mechanism for pH homeostasis involves urea amydolase Dur1,2, which enables *C. albicans* to use urea as a nitrogen substrate by converting it into ammonium, subsequently released from the cell, leading to environmental pH alkalinization [61]. Moreover, experiments on mice revealed that Dur1,2 plays a crucial role in *C. albicans* colonization of the kidney and brain, in host proinflammatory cytokines and chemokines response, and in regulation (over-expression) of inflammatory genes from the kidney [62].

In *Y. lipolytica*, pH sensing is mediated by Rim101p and four Pal1-4P proteins, which directly control alkaline (XPR2)/acidic (AXP1) serine proteases synthesis in near-neutral conditions, as well as mating and sporulation. Overall, for the members of this species, similar to *C. albicans*, proton extrusion and plasma membrane ATPase activity, coupled with modified mitochondrial function, represent the main pathways used for maintaining pH homeostasis. Thus, in terms of overall response to acidic pH, TCA cycle enzymes, respiratory quinones, and mitochondrial porins are upregulated to maintain the intracellular pH. Meanwhile, for alkaline pH, a strong decline in intracellular sugar and lipid content is observed [63]. Interestingly, for *Y. lipolytica*, apart from the Pma1p involvement in pH homeostasis, another approach is redirecting the carbon flux towards the accumulation of α-ketoglutarate, which acts as an intracellular buffer as well as an antioxidant to counteract acid-induced ROS stress (Figure 1) [64].

## 3. CO_2_ Sensing and Response to Oxygen Limitation

### 3.1. CO_2_ Exposure

*C. albicans* pathogenesis is promoted by the excess of CO_2_ (in hypercapnic states, exhaled breath) and hypoxia (in hyperventilation, reduced blood capacity to carry oxygen, perfusion-related problems, etc.) [65].

In the presence of elevated CO_2_ levels, carbonic anhydrase (CA) Nce103 plays an essential role in the pathogenesis of *Candida* cells. Nce103 is found in the cell wall, membrane, and in subcellular fractions (mitochondrial and cytosolic) of *C. albicans* and *C. parapsilosis* cells and represents a sensing receptor for signaling the CO_2_ levels. Nce103 is also responsible for catalyzing the conversion of CO_2_ into bicarbonate (HCO_3_) [66,67]. Further, the bicarbonate activates the Cyr1-PKA (protein kinase A) pathway, promoting the expression of biofilm transcriptional regulators Efg1, Brg1, Bcr1, and Ndt80 and hyphal development in *Candida* cells [68].

According to Cottier et al. [69], the carbonic anhydrase Nce103 is essential in sensing low environmental CO_2_ levels (0.03%) in *N. glabratus*. Its activity is regulated by the Rca1 transcription factor with a role in hyphal formation through the cAMP-PKA and Efg1 pathway. On the contrary, under high CO_2_ levels (5.5%), Nce103 function appears to be optional. The Sch9-Rca1-Nce103 CO2 sensing module, described as promoting β-1,3-glucan masking in *C. albicans*, is conserved in evolutionarily divergent yeasts and is also described for *C. krusei* and *C. tropicalis* [70,71].

A similar carbonic sensing pathway (CSP) was characterized for *C. auris* carbonic sensing pathway (CSP), using an integrated proteo-transcriptomics approach [72]. Furthermore, in this case, Nce103 also plays a key role in amphotericin resistance.

Lu et al. [65] showed that in the presence of double stress conditions represented by low oxygen (0.2%) and high CO_2_ (5%) concentrations, *C. albicans* cells form elongated, stable hyphae independent of nutrient limitation. The process involves blocking the synthesis of Ofd1 (prolyl hydroxylase) and regulating the production of Ume6, a transcriptional activator with a specific function in initiation and extension of hyphal filaments. Additionally, Ume6 acts in correlation with Efg1 and Ndt80 for the activation of genes involved in dimorphism and adherence. In a different experiment conducted at the same CO_2_ concentration (5%), the *C. albicans* biofilms showed increased drug resistance due to upregulation of the *MDR1* gene (multidrug resistance gene), as well as an enhanced tolerance to iron starvation [68].

Until present, the intracellular response to CO_2_ has not been investigated in *Y. lipolytica*, as this species appears to prioritize nutrient-derived stress rather than CO_2_ for regulating the morphological yeast–pseudohyphae transitions. In this respect, Liang et al. [73] described the activation of the TORC1-Sch9-Rim15 signaling pathway in response to nutrient conditions, underlining the shared components of this signaling pathway with its homologues from *C. albicans*. This indicates the necessity of further studies focused on the intracellular response of *Y. lipolytica* to elevated CO_2_ environments in relation to its opportunistic pathogenicity [73].

### 3.2. Oxygen Limitation. Hypoxia

In order to colonize low-oxygen niches (vagina or gastrointestinal tracts) as well as tissues with high levels of oxygen (skin epithelium or oral mucosa), *Candida* cells must adapt to environmental oxygen supply. This trigger modifications of central metabolism related to the overexpression of genes involved in glycolysis, hexose transport, fermentation, amino acids, and glycerol metabolism. The main genes upregulated in oxygen and hypoxia sensing are encoding transcriptional factors with role in specific pathways: Tye7 (glycolysis), Gal4 (galactose metabolism), Ahr1 (amino acid metabolism), Efg1 (dimorphism), and Upc2 (azole resistance and sterol synthesis), or specific proteins: Sit4 phosphatase (hyphal growth), Ccr4 mRNA deadenylase, and Sko1(cell wall stress signaling) [74,75].

In *C. albicans*, oxygen limitation correlates with iron metabolism. Thus, hypoxia determines upregulation of the genes *RBT5* and *PGA7*—encoding homophores with a role in iron acquisition from hem—and *PGA10* (*RBT51*)—encoding a plasma membrane protein with multiple functions in iron utilization and as an adhesion molecule [76]. The three proteins (Rbt5, Pga7, and Pga10) are part of the CFEM family (Common Fungal Extracellular Membrane domain-containing protein), mainly represented by glycosylphosphatidylinositol (GPI) proteins with roles in differentiation, stress response, and pathogenicity [76].

Burgain et al. [77] showed that under hypoxic conditions, the Snf5 protein from the SWI/SNF chromatin remodeling complex is required for adaptation of *C. albicans* metabolism to utilize alternative carbohydrates in the absence of glucose (such as galactose) as well as for survival both in commensal and invasive form. The effect of Snf5 seems to be correlated with its regulatory activity on Cyr1, part of the cAMP-PKA signaling pathway [78].

Recent studies revealed that a massive influx of polymorphonuclear leukocytes at the infection site generates hypoxia, creating an environment that triggers β-glucan masking from the cell wall surface. The process impairs the response of macrophage and neutrophils, reducing the activity of dectin-1-dependent myeloid cells, and favoring *C. albicans* colonization [79]. The mechanism involves the cAMP-PKA pathway and mitochondrial stress signaling genes *GOA1* (encoding a regulator for resistance to oxidative stress) and *UPC2* (encoding a zinc cluster transcription factor essential for ergosterol synthesis and azole resistance). Hypoxia-induced β-glucan masking is also indicated as a mechanism for other Candida species, mainly *C. tropicalis* and *C. krusei*, and some strains belonging to *C. parapsilosis* [80].

Metabolomics and transcriptomics studies [81] on *C. albicans* biofilms during their initial stage of formation (first 8 h of development) revealed that low oxygen levels have a strong impact on their metabolic network, including ATP metabolism and mitochondrial respiratory chain. The number of upregulated genes (741) exceeded those downregulated (488), referring to genes involved in carbohydrate transport > regulation of cell adhesion > cellular carbohydrate metabolic process > lysosomal microautophagy > biological adhesion > biofilm formation > cell aggregation > response to chemical > cell adhesion. The metabolic flux is subsequently restored following an 8 h adaptation period.

In *N. glabratus*, hypoxia induced the formation of biofilm, the relative reduction in metabolic activities (RRMA) in hypoxic biofilms being a maximum of 35% compared to 50% in normoxic biofilms. Under the same conditions, the genes *ROX1* (stress response regulator), *ECM33*, *KRE1*, and *KRE2* (with key roles in fungal cell wall synthesis and organization) seemed to be the most actively involved in biofilm formation [82].

If *C. albicans* is a facultative anaerobe and can undergo fermentation upon semi-anaerobic conditions from infection sites in the human gastrointestinal tract, kidney, and lungs, *Cryptococcus neoformans* is an obligatory aerobe, and its metabolism relies exclusively on mitochondrial respiration. In *C. neoformans*, exposure to low-oxygen conditions determines the formation of titan cells (up to 100 µm diameter) with a thicker cell wall and a dense capsule, which are resistant to phagocytosis and can produce normal-size budding cells responsible for spreading the infection in the organism. The molecular mechanism involves the transcription factor Sre1, an oxygen level regulatory sensor with a role in ergosterol synthesis, belonging to the SREBPs family (Sterol regulatory element binding proteins) [83,84,85]. On the contrary, in *C. albicans*, under hypoxic conditions, sterol synthesis is not mediated by members of SREBPs, but instead relies on Upc2 [78].

Besides Sre1, in *C. neoformans*, the Pas2/Rds2 transcription complex plays a key role in gene regulation in a hypoxic environment. The PAS (Per-Arnt-Sim) domain-containing proteins have a role in environmental conditions signaling in higher eukaryotes and may also be responsible for adaptation to low-level oxygen in fungi. In this respect, Zhao and Lin [86] demonstrated that the expression of genes involved in glycolysis and the TCA cycle was highly affected in *pas2Δ* mutants, and that Pas2 interacts with Rds2 (a regulator of drug sensitivity) in the nucleus, assuring a normal cryptococcal growth.

In contrast, *Y. lipolytica*, which is an obligatory aerobe yeast, exhibits adaptive intracellular responses under oxygen deprivation mainly through metabolic and transcriptional remodeling rather than through the designated hypoxia-sensing pathways. Overall, low levels of oxygen cause pseudohyphal transitioning in *Y. lipolytica* associated with a decrease in lipid metabolism initiated by downregulation of ATP-citrate lyase and malic enzyme and secretion of Krebs cycle intermediates such as citric acid [51,87]. The study of Timoumi et al. [88] showed that in batch cultivations, oxygen level fluctuation (between 0 to 40% pO_2_) causes yeast growth interruptions, with the growth rate declining fast from almost 0.24h^−1^ to zero. This is mainly due to the repression of the mitochondrial ADP/ATP carrier encoding gene *YlAAC1* [89]. However, Mentel et al. [89] point out that *Y. lipolytica* presents three *YlAAC* genes, of which only *YlAAC1* and *YlAAC2* seem to be constitutively expressed, since they are not significantly influenced by moderate metabolic shifts during normal growth. Thus, the expression of each *YlAAC* gene is weakly influenced by glucose or lactate/oleate and is reduced by the presence of proteins, such as casein, as the carbon source.

## 4. Thermotolerance Mechanisms

*C. albicans* cells have a high ability to adapt to temperature variations characteristic of the human body and to a febrile state. Heat shock determines transient acidification of the cytoplasm, required for activation of the *HSF1* gene and synthesis of Hsf1 (heat shock transcription factor 1). On this turn, Hsf1 controls genes encoding for chaperone proteins, proteins involved in ubiquitination, oxidative stress response, or the cAMP-PKA pathway. Moreover, Hsf1 also regulates genes involved in protein folding homeostasis as a mechanism of cell defense against the accumulation of reactive oxygen species and protein misfolding [90].

In *Candida* and other fungi, there are two classes of heat shock proteins: ATP-dependent high molecular—Hsp90, Hsp60, Hsp70, and Hsp104, and ATP-independent low molecular weight (12–42 kDa)—Hsp10, Hsp21, and Hsp30/31, called small heat shock proteins (sHSPs). Among these, the cell response to thermal stress is mediated by Hsp90, Hsp60, Hsp70, Hsp12, and Hsp21 [45].

Leach et al. [91] used chromatin immunoprecipitation (ChIP) of Hsf1-TAP followed by sequencing to identify the Hsf1 binding sites in the *C. albicans* genome under heat-shock conditions. They obtained 104 sites in total, 55 of them representing heat-shock-dependent targets. Under these conditions, Hsf1 upregulated the genes for heat shock proteins *HSP90*, *HSP21*, and *HSP104*, as well as the genes *ALS1*, *ALS3*, and *ROB1*, which are co-regulated by other transcriptional factors involved in filamentation, hyphal, and biofilm development. However, Hsp90 appears to be the main protein involved in *C. albicans’* response to thermal stress. In this matter, a particular case is represented by the regulatory loop formed by Hsf1-Hsp90. Thus, Hsf1 regulates the synthesis of heat-shock chaperon Hsp90 by binding, either as a trimer or a dimer, to the HSE (heat-shock element) sequence placed upstream of the gene promoter. Subsequently, in the short-term thermal adaptation process, Hsp90 represses Hsf1 action by chromatin remodeling (nucleosome positioning) and blocking the Hsf1 binding sites.

**Figure 2:**
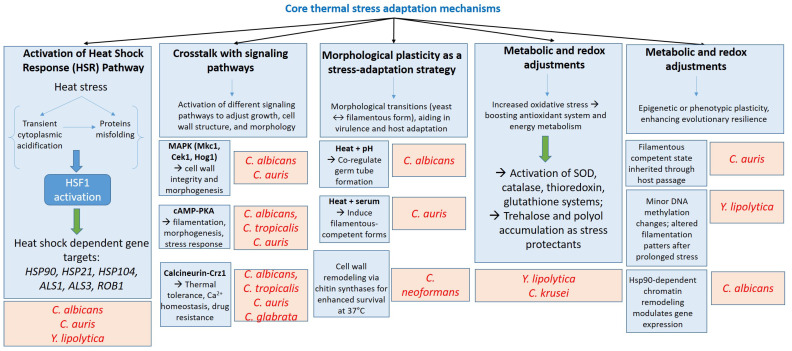
Integrated thermal stress response pathways linking heat shock, signaling, morphological plasticity, and metabolic adaptation in pathogenic fungi.

Chaperon Hsp90 also participates in long-term thermal adaptation of *C. albicans* cells, in correlation with the MAPK kinase-mediated pathways, by regulating the synthesis of Mkc1, Cek1, and Hog1 involved in cell wall integrity and biogenesis, respectively, in stress response and morphogenesis [91]. Moreover, according to Senn et al. [92] and O’Meara and Cowen [93], Hsp90 interacts with Cdc28 and regulates cyclin Clb4. Inhibition of Hsp90 reduces the levels of Clb4 and leading to delayed mitosis and increased filamentation (Figure 2).

Jara et al. [94] studied the whole genome sequences of 801 strains belonging to *C. auris* and found that Hsp90 and Hsp104 were present in over 98% of them. Hsp90 is essential for growth and azole tolerance, the latter being linked to inhibition of ergosterol synthesis. Surprisingly, deletion of the gene *HSP90* leads to polarized cell growth in a process similar to filamentation. In fact, the experiments conducted with modified strains bearing a compromised *HSP90* function showed that the effect of this mutation consisted of a relative abundance of filaments vs. yeast form cells and decreased in the following order: *C. dubliniensis* > *C. albicans* > *C. tropicalis* > *C. lusitaniae* > *C. auris* [95].

The cAMP-PKA signaling pathway also plays an important role in thermotolerance of *C. albicans* through two mechanisms. On one hand, two of the PKA subunits are involved in cellular response to high temperature (50 °C): the Tpk1 subunit confers cell sensitivity, while the Bcy1 subunit is essential for cell survival [96]. On the other hand, under basal conditions, Hsp90 physically interacts with Cyr1 through its co-chaperone Sgt1, blocking Cyr1 function and, consequently, the cAMP-PKA cascade. At elevated temperatures, Hsp90 prioritizes maintaining a correct protein folding process, releasing Cyr1, which can then activate the cAMP-PKA pathway for the expression of genes involved in filamentous growth associated with *Candida* pathogenesis [97,98] (Figure 2).

PKA is also involved in thermal stress response in *C. tropicalis*. In this respect, the mutant strains bearing deletions for both Tpk1 and Tpk2 subunits presented defective growth at extreme temperatures, specifically, 25 °C and 42 °C [99].

Temperature shift may act in combination with other stress conditions to influence *Candida* pathogenesis. Thus, Nadeem et al. [100] observed that 40% of *C. albicans* cells formed germ tubes after 4.7 h of incubation at 37 °C and in a neutral–slightly alkaline pH (7.4) medium, thus confirming a correlated action of temperature and pH over hyphal development.

The transition of *C. auris* cells to filamentous form in the presence of body temperature and blood serum determines *C. auris* transition to filamentous form mediated by the Cyr1-cAMP-PKA cascade. In comparison to *C. albicans*, where Cyr 1 is regulated by Gpr1, in *C. auris*, it interacts directly with Ras1 [101]. It is also interesting to notice that the Tpk1 and Tpk2 subunits of PKA have double roles in *C. auris* pathogenesis: cAMP-dependent (morphological transition and stress response) and cAMP-independent (transition from haploid to diploid state, chitin and chitosan synthesis, and biofilm formation) [102].

Although *C. auris* does not form filaments in vitro, existing only in yeast form, two other cell types have been described: filamentous competent (FC) and filamentous cells, respectively. The transition from yeast form to FC or filamentous morphology is heritable and is determined by the passage through the infected mammalian host. On the other hand, the transition between FC and filamentous phenotype is not heritable and is temperature dependent. Moreover, it seems that the filamentous type is favored by low temperatures (20–25 °C) [103].

The ability of *C. auris* to grow and produce virulence factors across a wide range of extreme temperatures is critical for its pathogenesis. Thus, *C. auris* produces SAPs both at a low temperature range and at 42 °C, which reflects the high thermotolerance of this species. However, SAPs determined at 37 °C in FC and filamentous cells showed more activity than in yeast cell forms, suggesting their possible role in virulence [12].

*C. auris* can adjust its growth at temperatures over 43 °C using the calcineurin signaling pathway. Calcineurin, a serine/threonine protein phosphatase composed of a catalytic subunit (Cna1) and a regulatory subunit (Cnb1), is activated by calcium/calmodulin (Cam1) in response to extracellular calcium and by Cch1/Mid1 channel in response to intracellular sources [101]. Calcineurin activates the Crz1 transcription factor, which, in turn, regulates genes involved in virulence, thermal, and drug tolerance.

The calcineurin pathway is also involved in thermal stress response in *N. glabratus*. In addition, Crz1 regulates genes for pH homeostasis and tissue colonization [32,104,105]. According to Liu et al. [106], *C. krusei* responds to heat shock through a reversible metabolic switch between glycerol and trehalose, regulated by the activities of GPD and trehalose-6-phosphate synthase, which assures cellular stability and recovery after thermal stress.

In *C. neoformans*, Cna1 is activated by Cam1 and plays an important role in growth at 37 °C. Under elevated temperatures, Cna1 targets a Crz1 ortholog; the Cna1-Crz1 pathway is required for expression of chitin synthase genes *CHS5* and *CHS6*, with a role in cell wall structure and fungal cell survival during infection [14]. Furthermore, Steinbach et al. [107] discovered that Cna1 also acts in correlation with the protein calcineurin temperature suppressor 1 (Cts1), and demonstrated that *C. neoformans cna1cts1* double mutants were nonviable (Figure 2).

*Y. lipolytica* has an optimal growth at 28–30 °C, while exposure to temperatures above 38 °C initiates accumulation of protective sugars such as trehalose, mannitol, or arabitol [108,109]. Heat stress also induces the remodeling of lipid composition both at the intracellular and membrane level, reduces sterol content, and increases the level of unsaturated fatty acid pools [109]. Exposure to thermal stress plays a role in the response to oxidative stress through upregulation of SOD^2−^, catalase Ctt1p, thioredoxin (Trx10), and accumulation of glutathione. These modifications are associated with increased content of heat-shock proteins and chaperones in order to stabilize the proteins [33,109]. Qiu et al. [110] and Wang et al. [111] proved that adaptive evolution or metabolic engineering performed by exposing *Y. lipolytica* strains to thermal stress or by overexpressing of *RSP5* gene from *S. cerevisiae* improves central carbon metabolism, amino acid biosynthesis, and ATP availability. In terms of epigenetic modifications, heat stress alone does not significantly alter DNA methylation, although it might reduce 5-methylcytosine levels and increase filamentation corresponding to long-term adaptive mechanisms [112]. Extreme temperature stress, causing dehydration, proved the protective role of trehalose in stabilizing protein and membrane integrity, whereas maintenance of cellular functionality in *Y. lipolytica* under thermal stress freezing has minimal impact [113]. Overall, these modifications enable *Y. lipolytica* survival and functionality under thermal stress.

## 5. Metabolic Adjustment to Nutrient Conditions

### 5.1. Carbon Substrates

*Saccharomyces cerevisiae* can use alternative carbon sources, such as galactose or lactose, as sole carbon substrates only in the absence of glucose, through glucose-regulated metabolic pathways. On the contrary, most pathogenic *Candida* species (*C. albicans*, *C. tropicalis*, and *C. parapsilosis*), and *N. glabratus* can simultaneously assimilate both glucose and alternative hexoses. In *C. albicans*, glucose induces hyphal formation, an important mechanism of pathogenicity that can lead to the death of macrophages depleted of their carbon substrate [1].

The main glucose sensor in *C. albicans* is Hgt4, which is identical to Snf3 (56%) and similar to Rgt2 (76%) from *S. cerevisiae* [114]. Hgt4 is sensitive to low glucose levels and induces the repression of *RGT1*. As a consequence, the transcription of the genes *HGT12*, *HXT10*, and *HGT7* (encoding hexose transporters) is activated. Furthermore, Hgt4 is involved in hyphal development: the *C. albicans hgt4Δ* mutants are mainly found in yeast form, whereas the *rgt1Δ* mutants presented hyperfilamentation [114,115,116].

*N. glabratus* responds to variations in glucose concentrations through different paths, depending on the glucose level. Thus, glucose concentrations between 0.01 and 0.1% boosted *N. glabratus* growth in vitro; a concentration of 2% was optimal for biofilm formation, while the range of 0.01–0.2% assured optimal resistance to amphotericin B, which correlates the glucose metabolism to virulence [117,118]. The main actor in sensing low levels of glucose is Snf3, which transfers the signal to yeast casein kinase Yck1 [117]. The activated Yck1 phosphorylates Std1/Mth1, which are recognized by the ubiquitin protein ligase complex SCF and further degraded by the proteasome. This inhibits the Rgt1 repressing activity, allowing the expression of *HXT* genes [119].

In *C. neoformans*, the hexose-like sensor Hxs1, a protein similar to Hgt4, is repressed by high glucose concentrations. However, this is essential for glucose uptake and cell growth under glucose limitations. Hxs1 plays an important role in fungal virulence and cell resistance to oxidative stress [120]. Low levels of glucose are essential for upregulation of *C. neoformans* antiphagocytic protein 1 (App1), a virulence factor with a major role in inhibition of cell internalization by macrophages.

Glycolysis is an important factor for *C. neoformans* virulence. In this respect, full functionality of genes encoding hexokinase 1 and 2 (*HXK1*, *HXK2*) and pyruvate kinase (*PYK1*) has been shown to be essential for cryptococcosis in murine and rabbit models [121,122].

The PKA-mediated cascade represents an important pathway used by *Candida* cells in response to variation in glucose levels, especially combined with other environmental factors. Studies on *C. albicans* demonstrated that assimilation of amino acids in the presence of glucose influences hyphal development through the G-protein-coupled receptor (GPCR) Gpr1, from which the signal is transmitted to the Gpa2 subunit of the G protein, to Cyr1, and, further, to the PKA pathway. On the other hand, the presence of glucose from the serum activates the Ras1-Cyr1-PKA pathway [68,123].

In vitro experiments showed that low concentrations of glucose (0.1%) activated the Cph1-mediated MAPK kinase cascade in *C. albicans*, and, more effectively, the Efg1-mediated cAMP-PKA cascade, promoting the activation of the genes involved in hyphae development and SAPs synthesis [124].

Growing *C. tropicalis* under low-level glucose (0.75%) in the presence of 10% serum at 37 °C induced overexpression of the Ume6 transcription factor within the first 0.5–1 h of exposure. This also triggered filamentation as part of the cAMP-PKA cascade, a characteristic essential for virulence and host colonization [125].

In the gastrointestinal tract, N-acetylglucosamine (GlcNAc) represents the main carbon substrate for several *Candida* pathogenic species, being a constituent of the cell wall of commensal bacteria. In this respect, in *C. albicans*, the GlcNAc sensor Ngt1 has a major role in upregulation of genes involved in GlcNAc uptake, catabolism (*DAC1*, *NAG1*, and *GIG1*), and filamentation (*HXK1*). The ability of *C. albicans* to bypass the morphogenetic transition from yeast to hyphae in the presence of GlcNAc under acidic pH conditions also enables the cells to escape destruction within the phagolysosome. In fact, the ability to neutralize acidic pH through the regulation of *DAC1*, *NAG1*, and *HXK1* is characteristic of other potential pathogenic *Candida* species within the CFG clade (the CUG codon is translated into serine instead of leucine) (*C. tropicalis*, *C. dubliniensis*, *C. lusitaniae*, *C. parapsilosis*). In contrast, the highly pathogenic species *N. glabratus* does not present GlcNAc metabolism [126].

The Ngt1 sensor also transfers the signal to Ngs1, activating histone acetylation and expression of genes involved in the transition from commensalism to pathogenesis. In this respect, GlcNAc was found to induce hyphae formation by Ngs1-mediated upregulation of *UME6*, which encodes the transcription factor Ume6 with a role in the development of hyphal filaments [127,128,129]. These findings were observed across several *C. albicans* strains (wild type and mutant) [129].

Zhang et al. [130] demonstrated that in *C. tropicalis*, the gene *HXK1* induces hyphae formation, phosphorylation of catabolism of internalized GlcNAc. On the other hand, in vitro experiments using medium supplemented with GlcNAc proved that the TPK pathway may also play a role in mycelium formation, with the *tpk2Δ* mutant cells presenting reduced hyphal development [99].

The less-studied *C. krusei* exhibits a broad environmental tolerance, minimal nutritional requirements, and versatile carbon metabolism, facts that enable its survival under harsh host conditions. The capacity to produce ethanol, acetoin, and phytase reflects a strong metabolic adaptability that may underpin its persistence and opportunistic pathogenicity [131]. Moreover, lysine succinylation represents a central regulatory mechanism linking metabolic activity to antifungal resistance. This post-translational modification alters the succinylation status of enzymes involved in glycolysis, gluconeogenesis, TCA cycle, fructose and mannose metabolism, and amino acid biosynthesis, promoting complex metabolic changes when exposed to amphotericin B. Comparative proteomic analyses revealed that AMB-resistant strains display aberrant glucose and amino acid metabolism, as well as enhanced ergosterol synthesis—collectively improving cellular tolerance to membrane-targeting drugs [132].

*Y. lipolytica* is widely known for its ability to assimilate a wide range of carbon sources, including hydrophobic substrates such as fatty acids, oils, or aliphatic hydrocarbons [133,134]. Unlike conventional yeasts (*S. cerevisiae*), *Y. lipolytica* can utilize simultaneously different substrates for growth without requiring complete repression of glucose assimilation [134]. This flexibility might facilitate *Y. lipolytica’s* ability to adapt to diverse host niches where nutrient availability is diverse, such as within phagocytes or mucosal surfaces. As an oleaginous yeast, *Y. lipolytica* redirects the metabolic flux toward lipid biosynthesis and storage when cultivated in nitrogen-limiting conditions and carbon source excess [135]. Lipid droplets serve both as an energy supply in a carbon-limited environment and as a protection mechanism against host immune defense mechanisms by providing substrate for membrane remodeling during host colonization. The growth curve of oleaginous microorganisms, such as *Y. lipolytica*, is influenced by the oleaginous growth phase, which starts after nitrogen depletion and continues until carbon exhaustion [136]. The transition between the growth and the lipid accumulation phases is regulated by the SNF1/AMPK pathway, which acts as a metabolic sensor monitoring the intracellular ATP levels. SNF1 negatively regulates the first committed step of fatty acid biosynthesis catalyzed by acetyl-CoA carboxylase, and any deletion or modification of SNF1 leads to increased lipid storage. Moreover, the *Y. lipolytica snf1* mutants show normal growth on glucose and glycerol but limited growth on oleate [137]. The study of Kerkhoven et al. [138] shows that the transcription profile of *snf1* mutants is not similar to the profile observed during oleaginous growth phase induced by nitrogen exhaustion, suggesting that its role is far from being well determined [138]. Even so, *SNF1* correlates cellular energy and carbon status through transcriptional and post-translational control, a fact that differentiates *Y. lipolytica* from *S. cerevisiae*. Consequently, *SNF1* represents a potential target for obtaining improved oleaginous phenotypes through genetic engineering [139,140]. It is important to emphasize that *SNF1* may enhance the yeast’s ability to tolerate stress and survive in host environments by linking energy status to lipid metabolism, thereby indirectly contributing to *Y. lipolytica*’s pathogenic potential during host colonization.

### 5.2. Nitrogen Substrate

The *C. albicans* genome presents two ammonium permease genes, *MEP1* and *MEP2*, from which the Mep2 protein plays a role in the induction of hyphal growth by transmitting the signal both to a MAP kinase and cAMP-PKA pathways similar to a GPCR [141]. The activity of Mep2 is regulated by GATA transcription factors Gat1 and Gln3, the last one being a major activator under nitrogen starvation conditions [142]. On the other hand, *C. albicans* cells release ammonium from amino acid metabolism, leading to pH alkalinization and induction of filamentous growth through Stp2 activity [56,116].

*C. neoformans* use urea for producing ammonium throughout urease synthesis, which increases the phagolysosomal pH and facilitates the spreading of cryptococcal infection [143].

In the host organism, the proteins are transformed into amino acids and peptides by the *C. albicans* SAPs. The sensor complex SPS (Ssy1, Ptr3, and Ssy5) plays a key role in sensing extracellular glutamine and arginine. Further, the signal activates the transcription factors Stp1 and Stp2. Stp1 regulates the expression of the gene *SAP2* encoding secreted aspartyl proteinases involved in virulence, biofilm formation, and filamentation in an acidic pH environment [144]. Meanwhile, Stp2 is required for expression of amino acid permease genes, environmental alkalinization, and hyphal development [81].

Methionine is essential for numerous molecular processes and is a precursor of S-adenosylmethionine (SAM) involved in nucleic acid methylation, tRNA modifications, biotin, and polyamine synthesis. In *C. albicans*, extracellular methionine uptake has a major role in yeast-to-hyphae translation. The process is mediated in a first step by Mup1, a high-affinity methionine permease and sensor. In the cell, methionine acts in correlation with S-adenosylmethionine, which is decarboxylated by Spe2 (SAM decarboxylase) to form polyamines, which further activate the cAMP-PKA cascade [145].

The G-protein-coupled receptor Gpr1, part of the Cyr1-cAMP-PKA pathway, also seems to be involved in amino acid sensing (especially methionine and proline) in the presence of glucose in *C. albicans* cells, although its precise role remains to be elucidated. However, in *C. neoformans*, a low level of methionine is sensed by Grp4 (similar to Gpr1), which activates hyphal growth through cAMP-PKA signaling [116,144].

Although *Y. lipolytica* is generally considered non-pathogenic, it shares some common nitrogen and amino acid-sensing pathways with pathogenic fungi. The members of this species possess three transcription factors, Gzf1p, Gzf2p, and Gzf3p, of which the last two are involved in nitrogen catabolism and lipid metabolism. Gzf3p acts as a general repressor of genes presenting GATAA motifs, while Gzf2p activates the genes involved in nitrogen assimilation. It is assumed that Gzf1p has the same role as Gzf2p. Overall, the nitrogen limitation is associated with the disruption of nitrogen regulators, leading to lipid accumulation. This process is possibly due to reduced expression of β-oxidation genes and also to indirect downregulation of genes presenting the carbon response element (GYGGGG) [146].

## 6. Conclusions

Understanding the molecular mechanisms that govern fungal pathogenicity under host-specific conditions is critical for unraveling the complex relationship between fungal pathogens and their human hosts. This review highlights both the conserved and species-specific strategies employed by *C. albicans*, NAC species, *N. glabratus*, *C. auris*, *Y. lipolytica*, and *Cryptococcus neoformans* to sense and adapt to environmental factors characteristic of different niches of the human body, such as pH and thermal variations, CO_2_ levels, hypoxia, and nutrient availability. While pathogens like *C. albicans* and *C. neoformans* exhibit well-characterized regulatory pathways that ensure their survival and spread, emerging pathogenic species such as NAC members, *N. glabratus*, *C. auris*, and *Y. lipolytica* are less studied, presenting a surprising and sometimes specific metabolic flexibility. The comparative study of these mechanisms is expected to offer a broadened and correlated insight into the molecular signaling pathways involved in their adaptation to human hosts, thus opening new perspectives for the discovery of antifungal targets and optimization of therapeutic strategies.

## Data Availability

No new data were created or analyzed in this study. Data sharing is not applicable to this article.
